# Supplementary material for: The Role of a “Treat-to-Target” Approach in the Long-Term Renal Outcomes of Patients with Gout
Source: J Clin Med. 2019 Jul 20;8(7):1067. doi: 10.3390/jcm8071067 (PMC6678146; doi:10.3390/jcm8071067)
Supplement: Supplementary file 1 [file jcm-08-01067-s001.pdf]

Supplementary Table S1. Characteristics of patients with moderate renal impairment at baseline.

Characteristics	Final serum urate ≥6 mg/dL (N = 8)	Final serum urate <6 mg/dL (N = 39)	*p*-value
Male sex	7 (87.5)	35 (89.7)	1.000
Age of ≥65 years	4 (50.0)	18 (46.2)	1.000
BMI* of ≥30 kg/m^2	0 (0)	2 (6.1)	1.000
Hypertension	6 (75.0)	24 (61.5)	0.692
Diabetes mellitus	1 (12.5)	4 (10.3)	1.000
ACEI/ARB† users	2 (28.6)	13 (44.8)	0.674
Diuretics† users	2 (28.6)	6 (20.7)	0.639
XOI users	6 (75.0)	24 (61.5)	0.692

Abbreviation: ACEI, angiotensin-converting enzyme inhibitor; ARB, angiotensin receptor blocker; BMI, body mass index; CKD, chronic kidney disease; XOI, xanthine oxidase inhibitor. Note: Except where indicated otherwise, values are expressed as number (%). * Data are available for 6 in subjects with final serum urate ≥6 mg/dL and 33 in subjects with final serum urate <6 mg/dL. † Data are available for 7 in subjects with final serum urate ≥6 mg/dL and 29 in subjects with final serum urate <6 mg/dL.

Supplementary Table S2. Overview of adverse events during the study period.

Events	Allopurinol users (N = 42)	Febuxostat users (N = 145)	Benzbromarone users (N = 57)	Overall (N = 244)	*p*-value
LFT abnormality	11 (26.2)	46 (31.7)	14 (24.6)	71 (29.1)	0.567
Dyspepsia	1 (2.4)	4 (2.8)	4 (7.0)	9 (3.7)	0.313
Diarrhea	0 (0)	3 (2.1)	1 (1.8)	4 (1.6)	1.000
Skin rash	0 (0)	5 (3.4)	3 (5.3)	8 (3.3)	0.459
AKI	3 (7.1)	3 (2.1)	2 (3.5)	8 (3.3)	0.201
Urolithiasis	2 (4.8)	6 (4.1)	1 (1.8)	9 (3.7)	0.715
Hematuria	0 (0)	1 (0.7)	0 (0)	1 (0.4)	1.000
UTI	0 (0)	1 (0.7)	0 (0)	1 (0.4)	1.000

Abbreviation: AKI, acute kidney injury; LFT, liver function test; UTI, urinary tract infection. Note: Values are expressed as numbers (%).
